# A more holistic view of the logarithmic dose–response curve offers greater insights into insulin responses

**DOI:** 10.1016/j.jbc.2024.108037

**Published:** 2024-11-29

**Authors:** Guanyu Wang

**Affiliations:** 1 Laboratory of Biocomplexity and Engineering Biology, School of Medicine, The Chinese University of Hong Kong, Shenzhen, China; 2 Futian Biomedical Innovation R&D Center, The Chinese University of Hong Kong, Shenzhen, China; 3 Ciechanover Institute of Precision and Regenerative Medicine, School of Medicine, The Chinese University of Hong Kong, Shenzhen, China; 4 Center for Endocrinology and Metabolic Diseases, Second Affiliated Hospital, The Chinese University of Hong Kong, Shenzhen, China

**Keywords:** logistic function, cumulative normal distribution function, dose-response, dose-effect, mathematical modeling

## Abstract

The stimulus-response curve is usually modeled by the Hill function due to its simplicity and clear molecular mechanisms (Michaelis-Menten type of kinetics). Unfortunately, the mechanisms do not explain why the stimulus is ubiquitously measured by logarithmic dose rather than the dose itself and why the log(dose)-response curve possesses such fine properties as symmetry and wide adjustability. Here, the dose-response is considered from a holistic perspective spanning multiple biological levels from molecules to the whole organism, which reveals that an appropriate model for log(dose) response is the cumulative normal distribution (CND) function, which had only statistical implication previously but now possess mechanistic-statistical duality. The present CND model establishes a connection between single-cell all-or-none responses and the graded response at the tissue/organism level, reveals the *raison d'être* of the logarithmic transformation, explains why log(dose)-response curve possesses many fine properties, and reveals new mechanisms of tissue/organism dose-response, including homogeneity-induced sensitivity. It also provides new insights into vital biological processes, such as the insulin dose-response.

A dose-response curve describes the relationship between the magnitude of response (denoted by *V*) and the dose of a stimulus (denoted by *X*) (Fig. 1*A*). When the horizontal axis is measured by the logarithm of the dose (denoted by *x*), the response curve *V*(*x*) is generally a smooth sigmoid curve (Fig. 1*C*) manifesting symmetry around a center point (the blue point), while the corresponding *V*(*X*) shows no such symmetry (Fig. 1*A*). Furthermore, in response to environmental or physiologic changes, *V*(*x*) often shifts in a parallel fashion along the horizontal axis (Fig. 1*E*). For example, an insulin response curve is generally a perfect sigmoid curve, which shifts parallel to the right during aging or during the development of obesity/diabetes, and the right-shift is regarded as a sign of insulin resistance (1, 2). Strikingly, this parallel shift vanishes immediately after *V*(*x*) is converted to *V*(*X*) through the substitution *x* = ln*X*. Figure 1*G* gives an example, where the 3 *V*(*X*) are apparently not parallel, but their corresponding *V*(*x*) in Figure 1*E* are parallel. Owing to these fine properties, the log(dose) is ubiquitously used to measure the stimulus when studying the stimulus-response relationship. However, the reasons are largely unknown why log(dose) is ubiquitous and why the logarithmic transformation leads to such fine properties. The current understanding is that the logarithmic transformation is sheer teleological—just to make the response curve elegant and handy.

**Figure 1:**
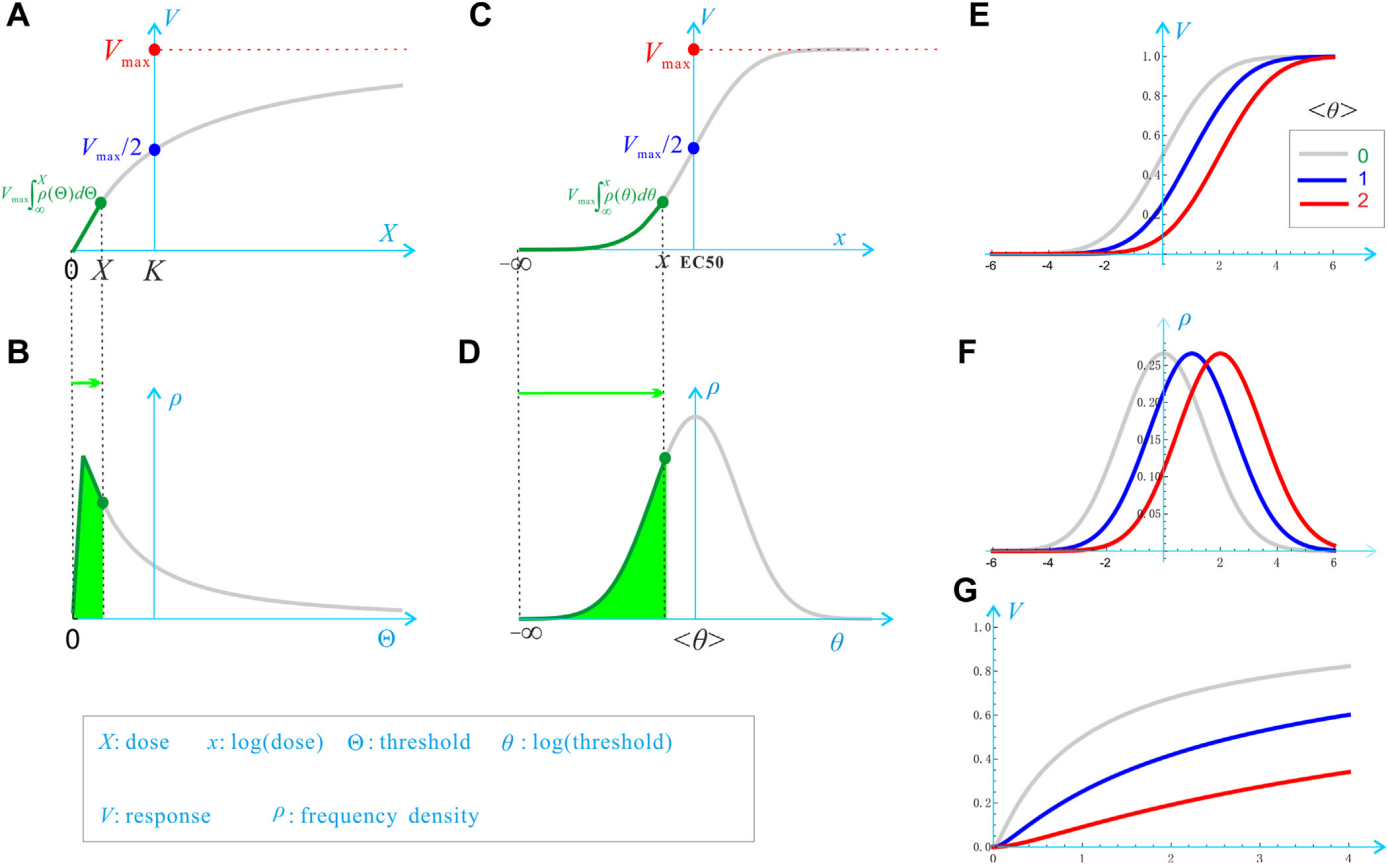
**The response of a tissue or whole-organism to a stimulus is the sum of the responses of cells of differential thresholds to the stimulus.***A*, the dose-response curve *V*(*X*) (Equation 12). *B*, the log-normal distribution of cells over the threshold values *ρ*(Θ) (Equation 13). The *green area* is precisely equal to the ordinate of the *green dot* in (*A*). *C*, the log(dose)-response curve *V*(*x*) (Equation 10). *D*, the normal distribution of cells over the log(threshold) values *ρ*(*θ*) (Equation 7). The *green area* is precisely equal to the ordinate of the *green dot* in (C). *E*, the parallel shift of 3 *V*(*x*), which are parameterized by two parameters <*θ*> and σ, with *σ* = 1.5 fixed and <*θ*> 0, 1, 2 for the *gray*, *blue*, and *red curves*, respectively. As <*θ*> increases, the curve shifts parallel to the right. *F*, the three *ρ*(*θ*) that underlie the 3 *V*(*x*) in (*E*). *G*, there is no parallel shift of *V*(*X*) as <*θ*> increases.

There are several mathematical functions that fit well with experimental log(dose)-response data (3), including the cumulative normal distribution (CND) function (4, 5, 6), the logistic function (7), the power law function (8), and the median-effect function (9). The use of CND to model dose-response has a long history dating back to the 1930s, which has only statistical implications. It was recognized that inherent variability in susceptibility to stimuli among individuals in a population results in a sigmoidal-shaped dose-response curve and that the response is some cumulation of the variability (5). However, the underlying cellular and molecular mechanisms have never been clear, and CND remains an empirical model. Consequently, the statistical explanation of dose-response is not well accepted, as exemplified by a claim in 2006 that “During the past 7 decades, evidence indicated that dose-effect analysis *per se* was a physicochemical rather than a statistical problem. In other words, it was deterministic rather than probabilistic” (3).

Among the deterministic models, the logistic function

(1) $$V\left(x\right)=\frac{V_{\max}}{1+e^{-h\left(x-k\right)}}$$

has dominated due to its simplicity, excellent fitting of experimental data, and clear molecular mechanisms. It is converted from the Hill function

(2) $$V\left(X\right)=\frac{V_{\max}}{1+{\left(X/K\right)}^{-h}}$$

by the logarithmic transformation *x* = ln(*X*) and *k* = ln *K*. The Hill function is based on the Michaelis-Menten type of kinetics of molecular binding (ligand-receptor or enzyme-substrate; for simplicity, I only mention ligand-receptor in the following), where *V*_max_ is the maximal reaction rate, *K* is the dissociation constant of the binding, and *h* is the Hill coefficient (which reflects cooperativity in the binding and determines the curve’s steepness). In theory, the Hill coefficient *h* is equal to the number of binding sites in the receptor. The greater *h* is, the more cooperative the binding, and the steeper the response curve. There are many biological examples of this relationship, the best known of which, formulated by Archibald Hill (10), is the binding of hemoglobin with four oxygen molecules (*h* = 4) (Fig. S1). Unfortunately, the Hill/logistic functions are limited to a single pair of molecular interactions and is removed from the immense cellular and molecular complexities that collectively shape tissue/organism dose response. In addition to the lack of mechanisms at the cellular level and beyond, statistical considerations are absent in the logistic function. These drawbacks notwithstanding, the logistic function remains the best-known model of dose-response owing to the advantages enumerated above.

Here, I have considered the dose response from a holistic perspective spanning multiple biological levels, from molecules to the whole-organism, which reveals both mechanistic and statistical implications of the dose-response and confirms that CND is an appropriate model for this analysis. Indeed, the tissue/organism response to a stimulus is the sum of the responses of many cells. The cellular heterogeneity, together with molecular fluctuations within a cell (see below), necessitates serious statistical considerations. For a given cell, the ligand-receptor binding triggers a cascade of molecular interactions, and the signal amplification culminates in the ultimate cellular response. The chemical reactions obey the law of mass action (LMA): the reaction rate is proportional to the product of the concentrations of the successive reactants in the chain of reaction (11). This multiplication of molecular concentrations is intrinsically awkward to express in a Cartesian abscissa-ordinate system, which is additive by design. Fortunately, this awkwardness can be removed by a logarithmic transformation on the concentrations because the multiplication of concentration is equivalent to the addition of log(concentration). Based on both a deterministic model of signal amplification and some standard statistical considerations, the reasonability is shown for using CND to model tissue/organism response, the mechanistic-statistical duality of dose-response is established, and the connection is established between single-cell all-or-none responses and the graded response at the tissue/organism level. In the following, I use uppercase and lowercase letters to indicate concentration and log(concentration), respectively. In particular, *X* and *x* denote dose and log(dose), respectively; *K* and *k* denote the dissociation constant on the dose and log(dose) scale, respectively.

## Results

### The mechanistic-statistical duality of the whole-organism dose-response

Chester I. Bliss realized that variability among individuals in their susceptibility to a stimulus leads to a cumulative response (5). An appropriate cellular/molecular mechanism embodying differential susceptibility is ultrasensitivity with different thresholds (12, 13, 14). Although exceptions do exist, threshold response holds true for many vital biological processes. For example, in response to molecularly targeted therapy, a cancer cell either conducts apoptosis or repairs the damage (15, 16); in response to a morphogen, a cell either maintains its original identity or turns into a new type of cell (17, 18); in response to insulin stimulus, a myocyte either takes up glucose all-out or abstains from glucose uptake, again an all-or-none response (14, 19, 20, 21); in response to steroid stimulus, the transcription in a single cell is either activated or not, and it occurs in bursts (22, 23, 24). The binary decision entails a threshold dose Θ of the stimulus, above or below which the cell gives a full or no response, respectively. This threshold can be used to quantify the sensitivity of a cell to the stimulus. The smaller Θ is, the more sensitive the cell is to the stimulus.

Consider an organism having *N* cells that are responsive to a stimulus *X* in an all-or-none manner, and assume that the cells are heterogeneous in their sensitivity to the stimulus; that is, different cells have different Θ values, forming a *frequency* distribution *ρ*(Θ) over the Θ-axis (Fig. 1*B*). When the stimulus is so large that all the *N* cells are activated, the whole-organism response reaches *V*_max_; namely, a single cell’s maximal response is *v*_max_ = *V*_max_/*N*. When the stimulus is at a smaller dose *X*, the whole-organism response is equal to $V_{\max}\int_{0}^{X}\rho \left(\Theta \right)d\Theta$, where the integral determines the frequency of the cells whose Θ values are smaller than *X*; namely, the green area in Figure 1*B*. As *X* increases, more and more cells become responsive, producing a graded response at the organismal level. Therefore, the key to understanding the dose-response curve lies in the obtaining of *ρ*(Θ).

**Figure 2:**
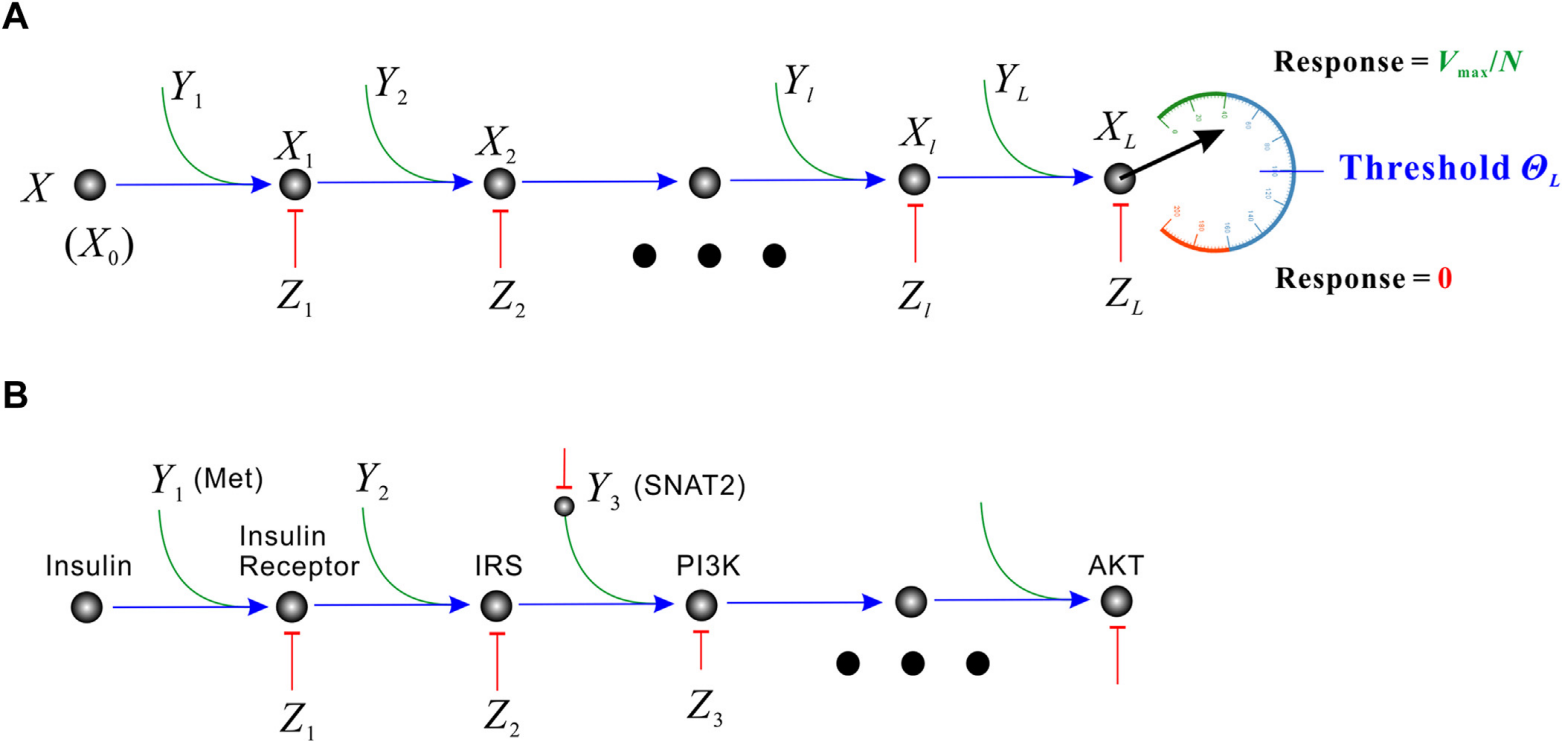
**The signaling cascade starts from the stimulus *X* and ends with the all-or-none response of a molecule *X***_***L***_**.***A*, a general representation. *B*, insulin signaling as an example.

Given that the organism is composed of innumerable cells, the law of large numbers (LLN) of probability theory tells us that the *frequency* distribution of many cells is equivalent to the *probability* distribution of a single-cell (25). Thus, one can infer the organism’s dose-response from the statistical properties manifested by a single cell. Figure 2*A* illustrates a cascade of signal transduction starting from the stimulus *X*, which is followed by a succession of transmitter molecules *X*_*l*_ (*l* = 1, 2, …, *L*) with *X*_*L*_ being an end point. A given transmitter *X*_*l*_ is subject to none, one, or several stimulators (denoted by *Y*_*l*_). That is, *Y*_*l*_ may represent just one stimulator, or multiple stimulators *Y*_*l*1_, *Y*_*l*2_, *Y*_*l*3_, ..., or it may be just void. As such, *X*_*l*_ may be subject to none, one, or several inhibitors (denoted by *Z*_*l*_). The end-point molecule *X*_*L*_ is compared to a threshold Θ_*L*_; only when *X*_*L*_ > Θ_*L*_, the cell produces a response *V*_max_/*N*. Note that Θ_*L*_ is in terms of the ending molecule *X*_*L*_, while Θ is in terms of the stimulus *X*; thus, Θ and Θ_*L*_ are called stimulus threshold and response threshold, respectively. Letting *θ*_*L*_ = ln(Θ_*L*_) and *θ* = ln(Θ) be the corresponding logarithmic thresholds, the whole signal transduction can be described by a set of *L* ordinary differential equations (ODEs) embodying plain mass action (26):

(3) $$\begin{array}{l} \frac{dX_{1}}{dt}=A_{1}Y_{1}X_{0}-B_{1}Z_{1}X_{1}\\ \frac{dX_{2}}{dt}=A_{2}Y_{2}X_{1}-B_{2}Z_{2}X_{2}\\ \vdots \\ \frac{dX_{l}}{dt}=A_{l}Y_{l}X_{l-1}-B_{l}Z_{l}X_{l}\\ \vdots \\ \frac{dX_{L}}{dt}=A_{L}Y_{L}X_{L-1}-B_{L}Z_{L}X_{L} \end{array}$$

where *A*_*l*_ and *B*_*l*_ (for *l* = 1, 2, …, *L*) are kinetic rates. Note that Equation 3 is valid when the pathway is “weakly activated,” that is, when all of the component kinases of the pathway are phosphorylated to a low degree (26). This is not a problem for the present theory because, under physiological conditions, most pathways are likely to be weakly activated (26); strongly activated pathways may exist and be sustained under certain pathological conditions, such as in a cancer cell (26). In the steady state, the left-hand sides of Equation 3 are all zero, which ultimately leads to the steady-state solution

(4) $$X_{L}=X\prod_{l=1}^{L}A_{l}Y_{l}/\prod_{l=1}^{L}B_{l}Z_{l}$$

Taking logarithm on both sides, one obtains

(5) $$x_{L}=x+\sum_{l=1}^{L}\alpha_{l}+\sum_{l=1}^{L}y_{l}-\sum_{l=1}^{L}\beta_{l}-\sum_{l=1}^{L}z_{l}$$

where the lower-case symbols represent the logarithm of their upper-case counterparts; *e*.*g*., *x*_*L*_ = ln(*X*_*L*_); *β*_*l*_ = ln(*B*_*l*_). Because the end-point response has been assumed all-or-none, *x*_*L*_ must exceed a response threshold *θ*_*L*_ to elicit the response, which renders a parallel behavior at the start side; namely, *x* has to exceed a stimulus threshold *θ* satisfying

(6) $$\theta =\theta_{L}-\sum_{l=1}^{L}\alpha_{l}-\sum_{l=1}^{L}y_{l}+\sum_{l=1}^{L}\beta_{l}+\sum_{l=1}^{L}z_{l}$$

according to Equation 5.

Each term on the righthand side (RHS) of Equation 6 can be considered as a random number because its value is different for different cells. More importantly, the central limit theorem (CLT) of probability theory states that *θ*, as the sum of random numbers, tends to follow the normal distribution, even if each RHS term is not normally distributed (27). Furthermore, even if the terms are non-identically distributed (28) or non-independent (29), *θ* still tends to be normally distributed in general (except for some extreme statistical conditions). The normal distribution is expressed by

(7) $$\rho \left(\theta \right)=\frac{1}{\sqrt{2\pi }\sigma }e^{-\frac{{\left(\theta -\left\langle \theta \right\rangle \right)}^{2}}{2\sigma^{2}}}$$

where *σ*^2^ is the variance of *θ*,

(8) $$\left\langle \theta \right\rangle =\left\langle \theta_{L}\right\rangle -\sum_{l=1}^{L}\left\langle \alpha_{l}\right\rangle -\sum_{l=1}^{L}\left\langle y_{l}\right\rangle +\sum_{l=1}^{L}\left\langle \beta_{l}\right\rangle +\sum_{l=1}^{L}\left\langle z_{l}\right\rangle$$

is the mean of *θ*, where the angle brackets represent mathematical expectation. Importantly, *ρ*(*θ*) can be regarded as both the probability distribution of *θ* of a single cell and the frequency distribution of *θ* of a population of *N* cells (Fig. 1*D*).

Based on the normal distribution of *θ*, the whole-organism response curve is determined by

(9) $$V\left(x\right)=V_{\max}\cdot \text{CND}\left(x\right)$$

Indeed, according to Figure 1*C*, the response at *x* is proportional to the fraction of cells activated by *x*, which is precisely the area under *ρ*(*θ*) and to the left of the vertical line *θ* = *x*:

(10) $$\text{CND}\left(x\right)=\int_{-\infty }^{x}\rho \left(\theta \right)d\theta =\frac{1}{2}\left[1+\mathrm{erf}\left(\frac{x-\left\langle \theta \right\rangle }{\sqrt{2}\sigma }\right)\right]$$

Note that the above analysis was on the log(dose) scale. On the dose scale, the response curve is a cumulative log-normal distribution (CLND) function:

(11) $$V\left(X\right)=V_{\max}\cdot \text{CLND}\left(X\right)$$

(12) $$\text{CLND}\left(X\right)=\frac{1}{2}\left[1+\mathrm{erf}\left(\frac{\ln\;X-\left\langle \theta \right\rangle }{\sqrt{2}\sigma }\right)\right]$$

and the corresponding frequency distribution function is the log-normal distribution over Θ:

(13) $$\rho \left(\Theta \right)=\frac{1}{\Theta \sqrt{2\pi }\sigma }e^{-\frac{{\left(\ln\;\Theta -\left\langle \theta \right\rangle \right)}^{2}}{2\sigma^{2}}}$$

Fig. S2 gives a more detailed explanation on the correspondence between the threshold distribution *ρ*(*θ*) (Fig. S2*A*), single-cell all-or-none responses *v*(*x*) (Fig. S2*B*), and the whole-organism dose-response *V*(*x*) (Fig. S2*C*).

In summary, the CND function is derived based on an ODE model of cellular signal transduction with the following simplifications: linear signaling cascade (no feedback/feedforward/crosstalk), weak interaction (low-degree of phosphorylation) satisfying LMA, all-or-none response with heterogeneous cellular thresholds, and the same magnitude of response once activated (*v*_max_).

### The CND model is still valid under more realistic conditions

The log(dose)-response is still approximately a CND function if a new model

(14) $$\frac{dX_{l}}{dt}=\frac{A_{l}}{1+{\left(K_{l}^{sti}/Y_{l}\right)}^{h}}X_{l-1}-\frac{B_{l}}{1+{\left(K_{l}^{inh}/Z_{l}\right)}^{h}}X_{l}$$

which is more realistic than Equation 3, is used. This model uses Hill functions to reflect saturation in the molecular interaction, where $K_{l}^{sti}$ ($K_{l}^{inh}$) are the half-maximal stimulation (inhibition) concentrations; *h* is a Hill coefficient. Equation 14 is also a standard model (see *e*.*g*., (30)), although more complex than Equation 3. The new model finally leads to a new threshold equation

(15) $$\theta =\theta_{L}+\sum_{l=1}^{L}\beta_{l}+\sum_{l=1}^{L}\ln\left[1+{\left(Z_{l}/K_{l}^{inh}\right)}^{h}\right]-\sum_{l=1}^{L}\alpha_{l}-\sum_{l=1}^{L}\ln\left[1+{\left(Y_{l}/K_{l}^{sti}\right)}^{h}\right]$$

**Figure 3:**
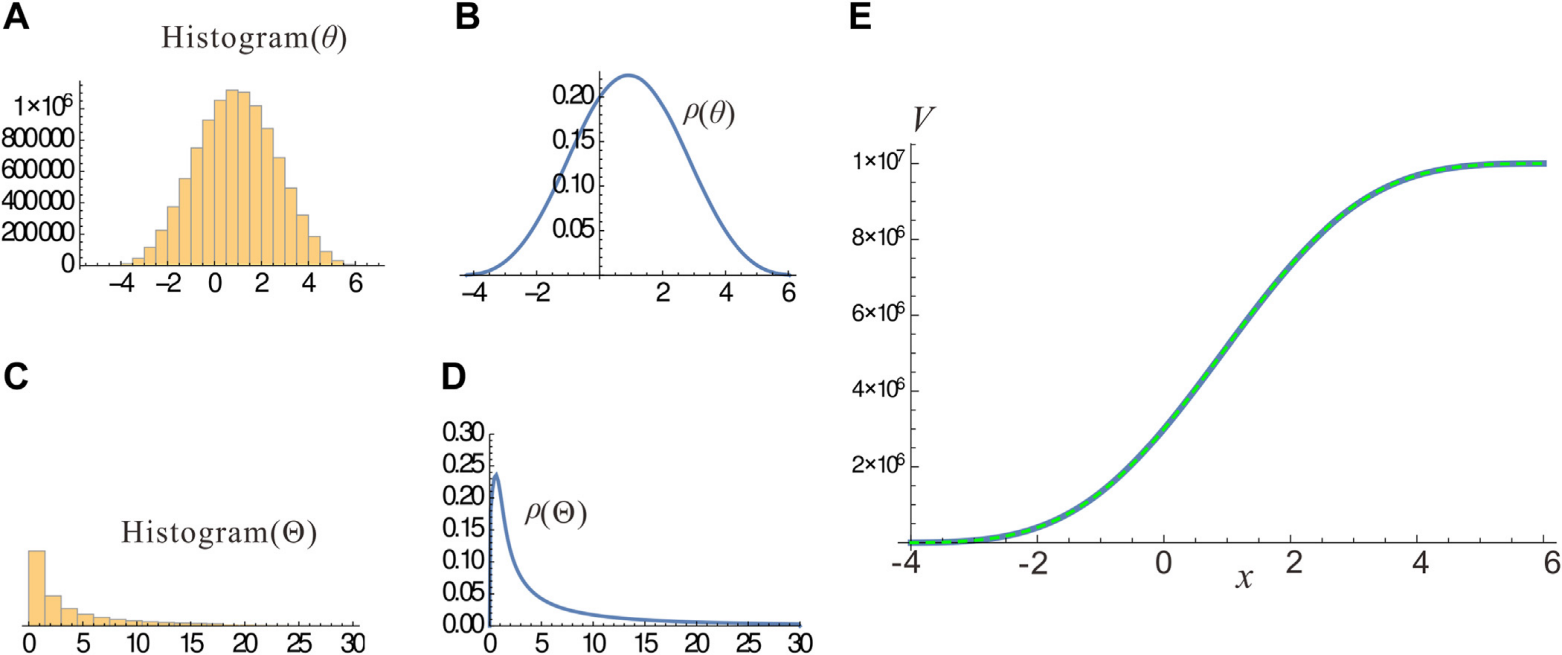
**The distribution of thresholds obtained by**Equation 15**from randomly (i.i.d.) generated *Y*, *Z*, α, and β values.***A*, the histogram of *θ*. *B*, the frequency density distribution *ρ*(*θ*). *C*, the histogram of Θ. *D*, the frequency density distribution *ρ*(Θ). *E*, the total response of the 10^7^ cells. The solid blue curve corresponds to the case that *v*_max_ = 1. The *dashed green curve* corresponds to the case that each *v*_max_ is a normally distributed random number with mean 1 and standard deviation 3.

The domination of summation in Equation 15 implies that *θ* should still be normally distributed. This supposition is verified by numerical simulation of Equation 15 with all the parameters fixed (Table 1) except *Y*_*l*_, *Z*_*l*_, *α*_*l*_, and *β*_*l*_ (*l* = 1, 2, …, *L*), each of which is an independent and identically distributed (i.i.d) random variable with minimum and maximum given in Table 2. In fact, Equation 15 is employed 10^7^ times to obtain 10^7^ values of *θ*. In each time, the *Y*_*l*_, *Z*_*l*_, *α*_*l*_, and *β*_*l*_ values are randomly drawn from their respective i.i.d. generators, after which the *θ* value is computed. I then generated the histogram (Fig. 3*A*) and the density distribution (Fig. 3*B*) for the obtained 10^7^ values of *θ*. For the corresponding 10^7^ values of Θ, the histogram (Fig. 3*C*) and density distribution (Fig. 3*D*) are also generated. Apparently, the *θ* and Θ values manifest normal and log-normal distributions, respectively, which is expected. The above results are obtained when the pathway is short (*L* = 2). If the pathway is longer, then there are more random parameters, and the distribution of *θ* would be even closer to normal because the validity of CLT enhances as the number of random variables increases. In summary, Equation 14 deviates from Equation 3 in two aspects: it is more realistic because the saturation of molecular interaction is taken into account, and the noise is i.i.d. thus deviating from normal distribution. Nevertheless, the log(dose)-response curve is still approximately a CND function, owing to the power of CLT.

**Table 1:** The values of the fixed parameters of Equation 15

L	h	*θ*_L_	*K*_1_^*sti*^	*K*_2_^*sti*^	*K*_1_^*inh*^	*K*_2_^*inh*^
2	1	0	6.9	2.1	4.0	6.1

**Table 2:** The minimal and maximal values of the i.i.d. of *Y*_*l*_, *Z*_*l*_, *α*_*l*_, and *β*_*l*_

Boundary	*Y*_1_	*Y*_2_	*α*_1_	*α*_2_	*Z*_1_	*Z*_2_	*β*_1_	*β*_2_
min	2.5	4.3	0.1	1.0	2.8	1.2	0.2	0.3
max	8.6	9.7	1.2	3.8	5.7	6.5	3.1	4.2

Figure 3*E* shows the log(dose)-response curve of the 10^7^ cells, where the solid blue curve corresponds to the case that *v*_max_ = 1 for all the cells (every cell has the same maximal response once activated); the dashed green curve corresponds to the case that *v*_max_ is a normally distributed random number with mean 1 and standard deviation 3 (the cells’ maximal response are all different). Even though the variance of *v*_max_ is large, the two response curves are almost identical, due to the averaging effect of the large number of cells.

### Logistic and CND functions fit equally well with experimental log(dose)-response data

**Figure 4:**
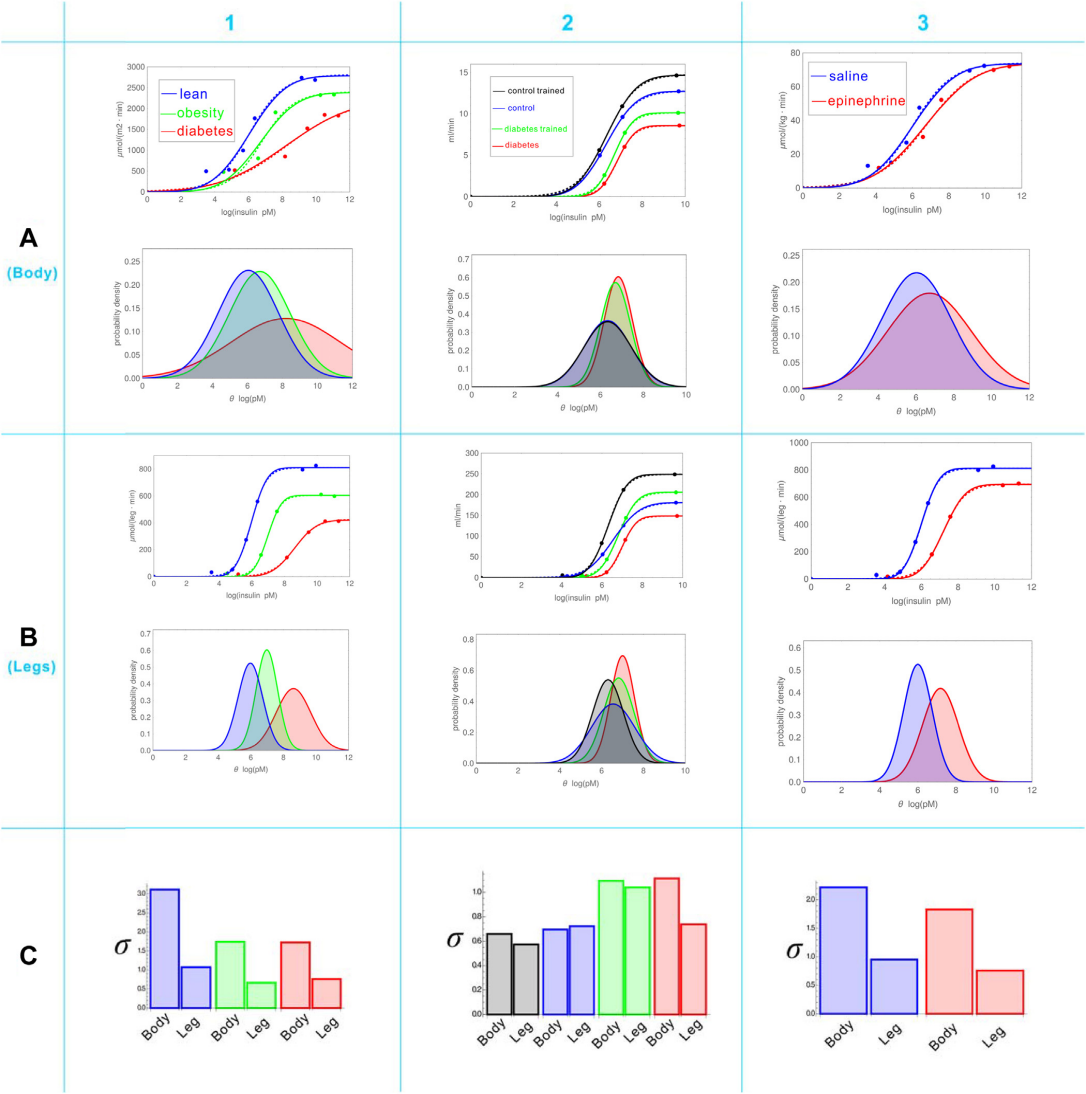
**Wide adjustability of efficacy and sensitivity in insulin-mediated glucose disposal dose responses of human subjects.***A*, Whole-body responses. *B*, leg-only responses. *C*, comparing *σ* between body and leg. (1) Research-based on the data from (45), where *blue*, *green*, and *red* represent lean, obesity, and diabetes subjects, respectively. (2) Research-based on the data from (37), where *blue* and *red* represent control and diabetes subjects, respectively; *black* and *green* represent control and diabetes subjects after physical training on the legs, respectively. (3) Research-based on the data from (44), where *blue* and *red* represent subjects treated with saline and epinephrine, respectively. The above classification divides the whole illustration into nine regions. Each region contains two figures: upper and lower. In each upper figure, the dots represent experimental data obtained from insulin clamp experiments; the *dotted (solid) lines* represent the curves generated by logistic (CND) functions that best fit the data. In each lower figure, the threshold distributions *ρ*(*θ*) are shown.

**Figure 5:**
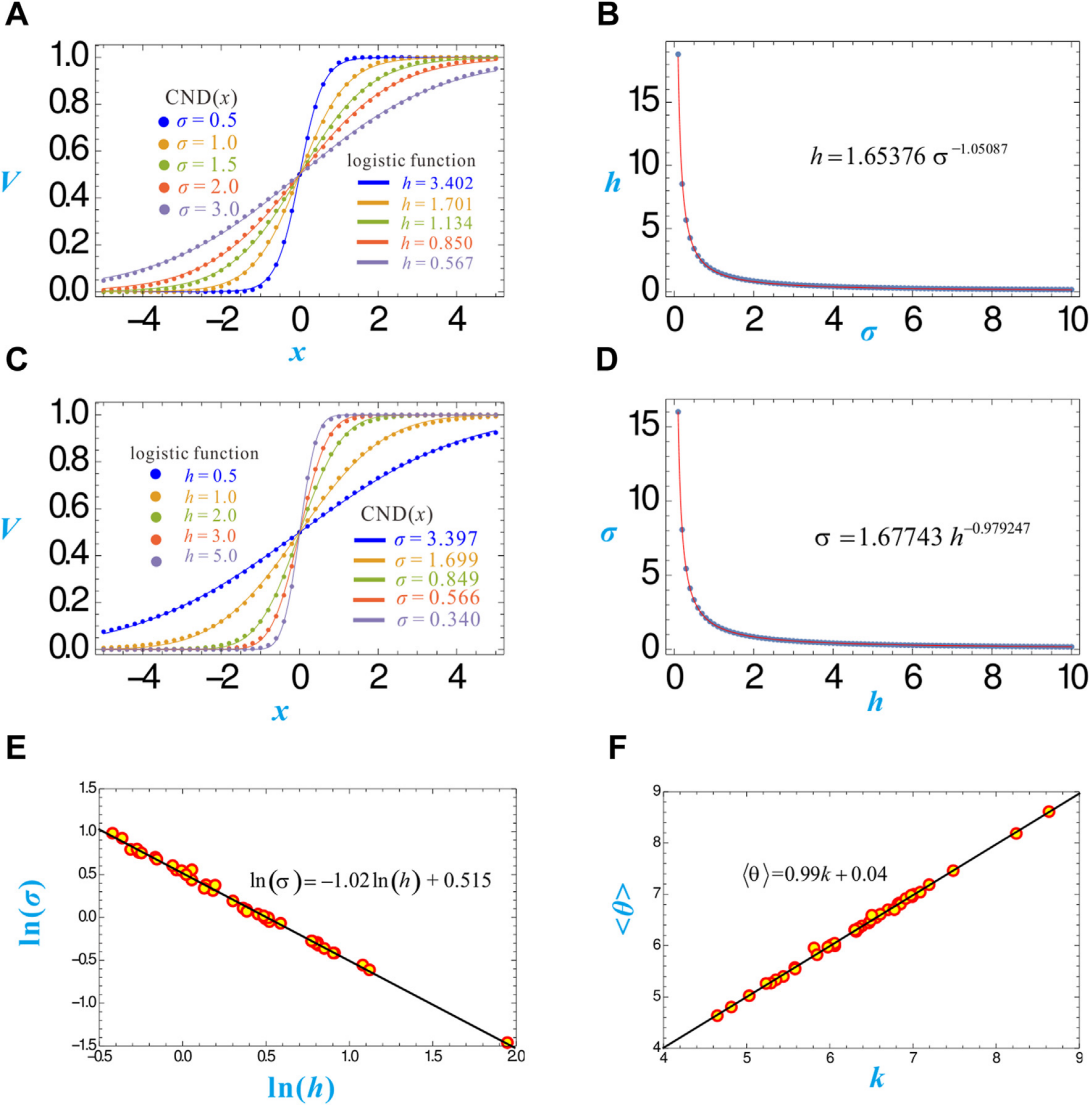
**The mutual fitting of CND function with logistic function.***A*, the *dotted curves* represent CND(*x*) with <*θ*≥ 0 and *σ* = 0.5, 1.0, … 3.0. The *solid curves* represent the logistic functions that fit optimally with the *dotted curves*, namely, with *k* = 0 and *h* = 3.402, 1.701, … 0.567. *B*, the 100 dots represent the 100 (σ, *h*) values obtained by the fitting described in (*A*). The *red curve* represents the power function that fits optimally with the 100 dots. *C*, the *dotted curves* represent the logistic function with *k* = 0 and *h* = 0.5, 1.0, … 5.0. The *solid curves* represent CND(*x*) that fit optimally with the dotted curves, namely, with <*θ*≥ 0 and *σ* = 3.397, 1.699, … 0.340. *D*, the 100 *dots* represent the 100 (*h*, *σ*) values obtained by the fitting described in (*C*). The *red curve* represents the power function that fits optimally with the 100 *dots*. *E*, the 42 dots represent the 42 (ln(*h*), ln(σ)) values obtained by fitting the experimental insulin dose-response data with the logistic and CND functions, respectively. The *solid line* represents the result of linear regression on the *dots*. *F*, the 42 *dots* represent the 42 (*k*, <*θ*>) values obtained by the same data-fitting as (*E*). The *solid line* represents the result of linear regression on the *dots*.

In Figure 4, the dots represent experimental data of insulin dose-response. They fit well by both logistic functions (dotted curves) and CND functions (solid curves). Strikingly, the two kinds of curves are almost indistinguishable, demonstrating equal effectiveness of the two models in fitting experimental data. More such examples are provided in Figs. S3 and S4. To see whether the equal effectiveness of data-fitting is accidental or not, I performed a mutual fitting between the two models and found that the simulated data generated by the CND function can always be fitted nearly perfectly by the logistic function (Fig. 5*A*), and *vice versa* (Fig. 5*C*). Therefore, equal effectiveness is not accidental but intrinsic. Taken together, it seems impossible to differentiate the two models based solely on the goodness of data fitting.

If the purpose of data-fitting is sheer teleological (*e*.*g*., to allow comparisons of different experimental conditions in a particular assay context), then both models are fine, and the logistic/Hill function, being simpler, is even more welcome. This explains why the Hill function is ubiquitously used to simulate biochemical processes, including the one in Equation 14 of the present paper, which is used teleologically to model saturation in molecular interaction. This teleological usage is sometimes under the name of *Hill function proper* (https://en.wikipedia.org/wiki/Hill_equation_(biochemistry)#cite_note-Hill1910-5).

### CND uncovers new mechanisms of whole-organism dose-response

Although teleology allows for the absence of mechanisms, it is always worthwhile, and sometimes important, to delve into mechanisms whenever possible. For example, the right-shift of insulin dose-response curve is closely related to the development of obesity and diabetes (19, 20); therefore, a mechanistic understanding of this right-shift would be beneficial to disease treatment.

To reveal the mechanisms, the correspondence between the response curve and model parameters needs to be found (Fig. S5). As shown in Fig. S5*A*, a response curve is characterized by *potency* and *sensitivity*. The potency is measured by the abscissa of the blue center point, which is precisely the half maximal effective concentration (EC_50_); it is determined by *k* of the logistic function and <*θ*> of the CND function. The sensitivity is measured by the slope at the blue center point; it is determined by *h* and *σ*. In other words, the horizontal shift of *V*(*x*) is controlled by *k* and <*θ*>, and the switch-likeness of *V*(*x*) is controlled by *h* and *σ*.

The logistic function, based on ligand-receptor binding, is not mechanistically applicable to tissue/organism responses. A tissue/organism dose-response involves the collective action of many molecules, which can hardly be considered by the logistic function. In particular, whole-organism dose-responses are often widely adjustable (*e*.*g*., the right-shift of insulin dose-response), whereas the parameters of the logistic function are largely fixed. In theory, *h* is equal to the number of binding sites in the receptor, which is largely fixed by the conformation of the molecules. The dissociation constant *k* is also determined by the molecular conformation; it may be affected by factors, such as temperature (the colder, the higher the affinity, and the smaller *k*), but the body temperature of a mammal is rather constant.

The CND function can naturally explain whole-organism response because it integrates biocomplexity of multiple levels including the organismal. The parameters of the CND function center on the thresholds, with <*θ*> representing their mean and *σ* quantifying their heterogeneity, which confers new understandings of dose-response. Firstly, it reveals that the potency (EC_50_) is determined by the mean threshold <*θ*>. Indeed, when *x* reaches <*θ*>, precisely half of the cells are activated (Fig. 1*D*), and the corresponding response is precisely *V*_max_/2 (Fig. 1*C*). Secondly, it reveals that the sensitivity of tissue/organism response is determined by the homogeneity of the constituent cells. The smaller *σ* is, the more homogeneous the thresholds, and the more sensitive the response. This homogeneity-induced sensitivity is a tissue/organismal level phenomenon, which is fundamentally different from molecular level sensitivity conferred by mechanisms, such as cooperative binding (31) and enzyme saturation (12, 13). This discovery is owing to the CND model.

In the absence of our holistic perspective and the CND model, the thresholds can hardly be detected because the dose-response curve may be very flat and show no sign of thresholds (*e*.*g*., the blue point in Fig. 1*A* does not look like a threshold at all, but it turns out to be a genuine threshold by a logarithmic transformation; see the blue point in Fig. 1*C*). Given that the logarithmic transformation is considered as a sheer teleological operation at present, the threshold is considered “pseudo,” for which the logarithmic transformation is blamed for causing an artifact of “visually implying a threshold dose when in fact there is none” (https://en.wikipedia.org/wiki/Dose%E2%80%93response_relationship). In light of the CND model, we now know that the logarithmic transformation is actually mechanistic, and the threshold is biologically meaningful.

Importantly, the parameters of the CND function are intrinsically variable, which makes it easy to explain the wide adjustability of the whole-organism dose-response curve. According to Equation 8, the mean threshold <*θ*> is the sum of many terms, which represent the many molecules in the signaling cascade, any of which can be targeted to cause the change in <*θ*>. According to Equation 7, this causes the horizontal shift of *ρ*(*θ*), which underlies the horizontal shift of *V*(*x*). For similar reasons, *σ* is also intrinsically variable. Suppose the fluctuations of the terms on the RHS of Equation 6 are independent, then *σ*^2^ is the sum of the variances of the RHS terms; therefore, any change in randomness of any molecule in the cascade will change the value of *σ*, which in turn changes the steepness of the response curve.

### Insulin response

It is well-known that the whole-body insulin log(dose) response is a simple graded curve (32), which belies the complexity of the single-cell response, which was recently demonstrated to be an all-or-none event using Akt phosphorylation as an indicator (14, 21). When insulin binds to the insulin receptor, it leads to a cascade of cellular processes including the successive phosphorylation of prominent regulators, such as insulin receptor substrate (IRS), phosphoinositide 3-kinase (PI3K), and Akt (Fig. 2*B*), ultimately leading to important biological functions, such as cellular glucose utilization and storage.

During aging, or the development of obesity and diabetes, the whole-body insulin response generally shifts to the right, and the right-shift is regarded as a sign of insulin resistance and is construed as a defect of insulin receptor function (1, 2). This reasoning depends on the fact that only a small fraction of receptors bind insulin at any given time, and the defects of insulin receptors can be overcome by overproducing insulin to bind more receptors, manifesting as the parallel right shift of the response curve. Although this sounds reasonable, there is no evidence for insulin receptor defects. With the CND model, which is widely adjustable, one does not have to seek insulin receptor defects to explain the shift. From Equation 6 one sees that any increase in inhibitor activity (*z*_*l*_) and/or the decrease in stimulator activity (*y*_*l*_) leads to an increase in <*θ*>, which can be easily achieved. For example, IRS is the second transmitter (*x*_2_) because it immediately follows the insulin receptor (Fig. 2*B*). Therefore, the inhibition of IRS makes *z*_2_ greater, which in turn makes <*θ*> greater (Equation 8), predicting the parallel right-shift of the insulin dose-response curve, which is exactly what happens (see Figs. 1 and 2 of (33)). PI3K, immediately downstream of the IRS, is a hub of regulation. For example, SNAT2 plays the role of *y*_3_ (amplifying PI3K activity), and the inhibition of SNAT2 is precisely the decrease in *y*_3_, which increases <*θ*> (Equation 8) and shifts the insulin dose-response curve to the right, which was verified by an experiment (see Fig. 3 of (34)). The insulin receptor, as *x*_1_, is also subject to regulation. For example, the stimulator *y*_1_ could be a hepatocyte growth factor receptor (Met), which stimulates insulin receptors by forming a hybrid complex, culminating in a robust signal output (35). These insights not only explain the naturally occurring right-shift of the insulin response curve but also suggest the therapeutic potential of targeting the insulin signaling pathway to treat obesity and type 2 diabetes (T2D).

Figures 4, S3 and S4 summarize 42 sets of experimental data on human whole-body insulin dose-response reported in the literature (32, 36, 37, 38, 39, 40, 41, 42, 43, 44, 45, 46, 47, 48, 49), where insulin concentration is shown with the unit ln(pM). Fig. S3 highlights the change in potency and suggests that the change may be caused by the shift of *ρ*(*θ*). In the upper panel of Fig. S3*A*, the red and blue dots represent experimental data of obese and control subjects, respectively (32); the solid and dotted curves are generated by the CND and logistic functions, respectively, that fit best with the data; the latter, being obscured by the former, are almost indiscernible. In the lower panel of Fig. S3*A*, the two ρ(*θ*) corresponding to the two CND curves in the upper panel are illustrated. The obese subjects’ ρ(*θ*) is to the right of that of the control subjects, with <*θ*> increasing from 5.573 to 6.386. In Fig. S3*B*, the red, green, and blue dots represent experimental data of upper obese, lower obese, and non-obese subjects (47), respectively. As expected, the order of ρ(*θ*) from left to right is non-obese (<*θ*> = 4.804), lower obese (<*θ*> = 5.264), upper obese (<*θ*> = 5.956), which implies that the lower obese is less healthy than non-obese but much healthier than the upper obese. In Fig. S3*C*, the red and blue dots represent experimental data of elderly and young men, respectively (42). The CND analysis reveals that the *ρ*(*θ*) of elderly men (mean age = 75.8) is to the right of the young men (mean age = 25.4), with <*θ*> increasing from 5.400 to 6.593. Although it appears that aging causes the right-shift of *ρ*(*θ*), the possibility exists that the right-shift is actually caused by fat gain, because elderly men (mean BMI = 26.2) have a significantly higher BMI than young men (mean BMI = 22.5). In Fig. S3*D*, the red and blue dots represent experimental data from individuals with hypertensive obesity and those who are normotensive with obesity, respectively (38). The CND analysis reveals that the *ρ*(*θ*) of the participants with hypertensive obesity is to the right of that of the normotensive individuals with obesity, with <*θ*> increasing from 6.811 to 7.042. The right-shift should be caused by hypertension because the BMIs of the study participants are comparable. Therefore, it can be concluded that hypertension exacerbates insulin resistance. In Fig. S3*E*, the red, green, and blue dots represent experimental data from individuals with hypertensive obesity, those after 6 months of aerobic exercise training plus weight loss, and normal lean controls (39). As expected, the order of *ρ*(*θ*) from left to right is normal lean (<*θ*> = 6.445), the treated hypertensive obese (<*θ*> = 6.953), the untreated hypertensive obese (<*θ*> = 7.462). In (49), normal weight healthy study participants were trained by moderate-intensity cycle exercise and then underwent recovery. Their insulin dose-response data are presented by the red (untrained), blue (trained), and green (recovered) dots in Fig. S3*F*. As expected, the order of *ρ*(*θ*) from left to right is the trained (<*θ*> = 4.636), the recovered (<*θ*> = 5.025), the untrained (<*θ*> = 5.824).

In Figure 4, the insulin response curves of the whole-body (Fig. 4*A*) and the legs (Fig. 4*B*) are compared. In terms of the shift of *ρ*(*θ*), the body and legs do not have essential differences. For example, Figure 4, *A1* and *B1* show the insulin response of the whole-body and the legs, respectively, of individuals with leanness (blue), obesity (green), and diabetes (red) (45); both have the same order of *ρ*(*θ*) from left to right: lean → obese → diabetic, indicating the strengthening of insulin resistance as the disease progresses. Figure 4, *A2* and B2 show the insulin response of the whole-body and the legs, respectively, of controls (blue) and individuals with diabetes (red) with both groups also undergoing physical training on the legs; the trained control and diabetes groups are indicated by black and green colors, respectively (37). The order of legs’ *ρ*(*θ*) (the lower panel of Fig. 4*B2*) is expected: trained control → untrained control → trained diabetes → untrained diabetes. The bodies’ *ρ*(*θ*) (the lower panel of Fig. 4*A2*) have almost the same order except that the trained control and untrained control have essentially the same *ρ*(*θ*) (compare the black *ρ*(*θ*) with the blue *ρ*(*θ*) in Figure 4*A2*). This equality is not surprising considering that the training on the legs may not exert significant influences on the whole-body, especially when the controls are already healthy. For the individuals with diabetes, however, the leg training did perceivably reduce whole-body insulin resistance, as demonstrated by the fact that the green *ρ*(*θ*) is slightly to the left of the red one in Figure 4*A2*. Figure 4, *A3* and *B3* describe the insulin responses of participants who have undergone epinephrine and saline infusion, respectively (44). For both body and leg, the *ρ*(*θ*) of epinephrine-treated participants is to the right of that of the saline-treated group, demonstrating that epinephrine may exacerbate insulin resistance.

Importantly, Figure 4 strongly supports the CND model. By comparing *ρ*(*θ*) between body and leg, one sees clearly that *ρ*(*θ*) of the legs are significantly narrower than those of the body, which can be easily explained by the simple fact that the legs are more homogeneous than the body. To be more quantitative, the *σ* value of each *ρ*(*θ*) is calculated and shown in the bar charts (Fig. 4*C*). The colors in Figure 4*C* correspond to those in Figure 4, *A* and *B*. Every color applies to a pair of bars, with the left (right) one corresponding to body (legs). For example, the left (right) red bar in Figure 4*C1* is used to express *σ* = 1.726 (0.761) for the *ρ*(*θ*) of body (leg) of the individuals with diabetes (45). For all the eight pairs of bars except the blue one in Figure 4*C2*, the bodies’ *σ* are higher than the legs’ *σ*. The obvious reason is that cells of the leg are more homogeneous than those of the body, so they have much narrower *ρ*(*θ*); namely, much smaller *σ* values. This implies that the CND model is more plausible than the logistic model.

Fig. S4 includes insulin dose responses not showing apparent shift characteristic (40, 41, 43, 46, 48).

### The approximately inversely proportional relationship between σ and h

When using the logistic and CND functions to fit the same set of experimental data, the estimated values of *h* and *σ* are quite different. Because they both correspond to the sensitivity of the response curve, it is worthwhile to determine the quantitative relationship between *h* and *σ*. This problem is addressed by first generating simulated data from the CND function with <*θ*> = 0 and a given value of *σ*. For example, the blue dots in Figure 5*A* represent the simulated data generated by a CND function with *σ* = 0.5. Then, the simulated data are fitted with a logistic function with *k* = 0, whereby the value of *h* achieving the best fitting can be found. For example, the blue dots in Figure 5*A* are best fitted by the blue curve, which is generated by the logistic function with *k* = 0 and *h* = 3.402. In this way, a correspondence between *σ* = 0.5 and *h* = 3.402 is found. Although only five examples (*σ* = 0.5, 1.0, 1.5, 2.0, 3.0) are shown in Figure 5*A*, I actually enumerated 100 values of *σ* ranging from 0.1 to 10 with a step size 0.1 and found their corresponding *h* values, which are represented by the 100 dots in Figure 5*B*, which suggest an inverse proportional relationship between *h* and *σ*. This supposition is tested true by fitting the 100 dots with a power function, which reveals that *σ*^1.05^ ∙ *h* ≈ 1.654. Reciprocally, simulated data are generated by the logistic function and fitted by the CND function (Fig. 5*C*), which result in 100 (*h*, *σ*) pairs (Fig. 5*D*), from which *σ* ∙ *h*^0.979^ ≈ 1.677 is revealed.

Given that the 42 sets of experimental data of insulin response (Figs. 4, S3 and S4) have been fitted by both logistic and CND functions, the 42 estimated (*σ*, *h*) pairs are used to further seek the *h versus σ* relationship, which reveals that *σ*∙*h*^1.02^ ≈ 1.682 (Fig. 5*E*). Together with Figure 5, *B* and *D*, I have demonstrated that *h* and *σ* are approximately inversely proportional satisfying *σ* ∙ *h* ≈ 1. 7.

The 42 estimated (<*θ*>, *k*) pairs are used to seek the *k versus* <*θ*> relationship, which reveals, not surprisingly, that <*θ*> ≈ *k* (Fig. 5*F*).

## Discussion

The log-normal distribution was discovered by Robert Gibrat, who, by considering CLT in the log domain (Gibrat’s law), found that the distribution of firm size is log-normal (50). Gibrat’s law is also applied to cities size and growth rate, where a proportionate growth process may give rise to a distribution of city sizes that is log-normal (51). Bliss developed similar insights during his study of the percentage of a pest killed by a pesticide (5). He perceived the statistical nature of dose-mortality relationship, realized the necessity of working on the logarithmic scale, and actually used the CND function to model the log(dose)-response curves. Due to a lack of biological mechanisms, however, the CND function remains as an empirical model and is little-known. The use of log(dose) is also empirical, with no mechanisms provided.

In this paper, the CND function has been considered from a holistic perspective and has been endowed with biological mechanisms (cell signaling pathway, Michaelis-Menten type of kinetics, LMA, signal amplification, ultrasensitivity). Together with its inherent statistical properties (threshold heterogeneity, LLN, CLT), this establishes a mechanistic-statistical duality for dose-response. The threshold heterogeneity arises from many variations in the cell, particularly the molecules along the signaling cascade. The successive biochemical reactions between the molecules follow the LMA, which ultimately leads to a combined strength proportional to the product of molecular activities. Because the abscissa is additive, a logarithmic transformation must be performed to convert multiplication into addition. Because of CLT, the *θ* values form into a normal distribution *ρ*(*θ*); thus, *V*(*x*), as the integration of *ρ*(*θ*), must be a CND function, and its symmetry originates from that of *ρ*(*θ*). As such, the parallel shift of *V*(*x*), which is one aspect of the wide adjustability of the tissue/organism response curve, can be explained by the parallel shift of *ρ*(*θ*) (Fig. 1*F*) and further by Equation 8. These results have confirmed that CND is an appropriate mathematical model of tissue/organism dose response. It has provided further insights into vital biological processes such as insulin responses, which in turn demonstrate the plausibility of the CND model, which can easily explain the sensitivity difference between body and leg. Together with the mechanistic-statistical duality, this implies that the CND function is a more suitable model than the logistic function.

## Experimental procedures

### Data acquisition

To test the validity and practicality of the CND model, I collected experimental data of human whole-body insulin dose response for the data-fitting by the CND model, as well as by the logistic model. There are not too many such data in the literature because their acquisition entailed euglycemic-hyperinsulinemic clamp experiments (32), which are time-consuming, poorly tolerated, and unhealthy. With the advent of HOMA-IR as a simple and handy method to assess insulin resistance (52, 53), the use of clamp technology to obtain insulin dose-response gradually decreased, resulting in almost no such data being published since the beginning of the current century. Through PubMed, I searched key words such as “insulin dose response” and “insulin clamp” and obtained dozens of publications recording human data of whole-body insulin dose-response. Many data were unusable due to too few data points. Finally, I collected 42 sets of human data from (32, 36, 37, 38, 39, 40, 41, 42, 43, 44, 45, 46, 47, 48, 49). They are shown as dots in Figs. 4, S3 and S4.

### Software and computation

All computations were performed using the software *Wolfram Mathematica* (WM) deployed on a Dell workstation (Precision Tower 7910). The validity of CND model was tested by using the threshold solution (Equation 15) of a realistic signal transduction model (Equation 14) to compute 10^7^ stochastic *θ* values, whose histogram and distribution curve, shown in Figure 3, *A–D*, were generated by packages Histogram[∙] and SmoothHistogram[∙], respectively, of WM.

In Figs. 4, S3 and S4, the fitting of experimental data was realized by using WM’s package.

NonlinearModelFit[*data*, *function*, *parameter*, *variable*].

Here

*data* is the set of experimental data in the form of ((*x*_1_, *V*_1_), (*x*_2_, *V*_2_), …). For example, the blue dots in Figure 4*A1* are represented by *data* = ((0, 0), (3.484, 493.1), (4.835, 532.9), (5.672, 990.3), (6.364, 1761.9), (9.144, 2739.0), (9.942, 2684.78)).

*function* is the *V*(*x*) used to fit *data*. It is either the logistic model (Equation 1) or the CND model (Equation 9).

*parameter* is the set of to-be-estimated parameters of *function*. It is either (*V*_max_, *k*, *h*) or (*V*_max_, <*θ*>, *σ*).

*variable* is the independent variable of *function*. It is simply *x*.

To accelerate the solution process, the ranges of the to-be-estimated parameters should be specified. These ranges are treated as generalized functions and thus included in *function* (not in *parameter*). For example, when I used the CND model to fit the blue dots in Figure 4*A1*, the *function* argument of NonlinearModelFit was as follows:

$$\mathrm{function}=\left\{\frac{V_{\max}}{2}\left(1+\mathrm{erf}\left(\frac{x-\left\langle \theta \right\rangle }{\sqrt{2}\sigma }\right)\right),2500<V_{\max}<3000,0.3<\sigma <4,4<\left\langle \theta \right\rangle <10\right\}\text{.}$$

By running NonlinearModelFit with the above specifications, the parameters were estimated to be: *V*_max_ = 2785.8, <*θ*> 6.056, and *σ* = 1.726, which gave the solid blue curve in Figure 4*A1*. The dotted blue curve can be similarly generated by using the logistic function to fit the data.

## Data availability

All data are contained within the manuscript.

## Supporting information

This article contains supporting information (32, 38, 39, 40, 41, 42, 43, 46, 47, 48, 49).

## Conflict of interest

The authors declare that they have no conflicts of interest with the contents of this article.
