# Supplementary material for: A more holistic view of the logarithmic dose–response curve offers greater insights into insulin responses
Source: J Biol Chem. 2024 Nov 29;301(1):108037. doi: 10.1016/j.jbc.2024.108037 (PMC11731574; doi:10.1016/j.jbc.2024.108037)
Supplement: Supplemental Fig. S1 [file mmc1.docx]

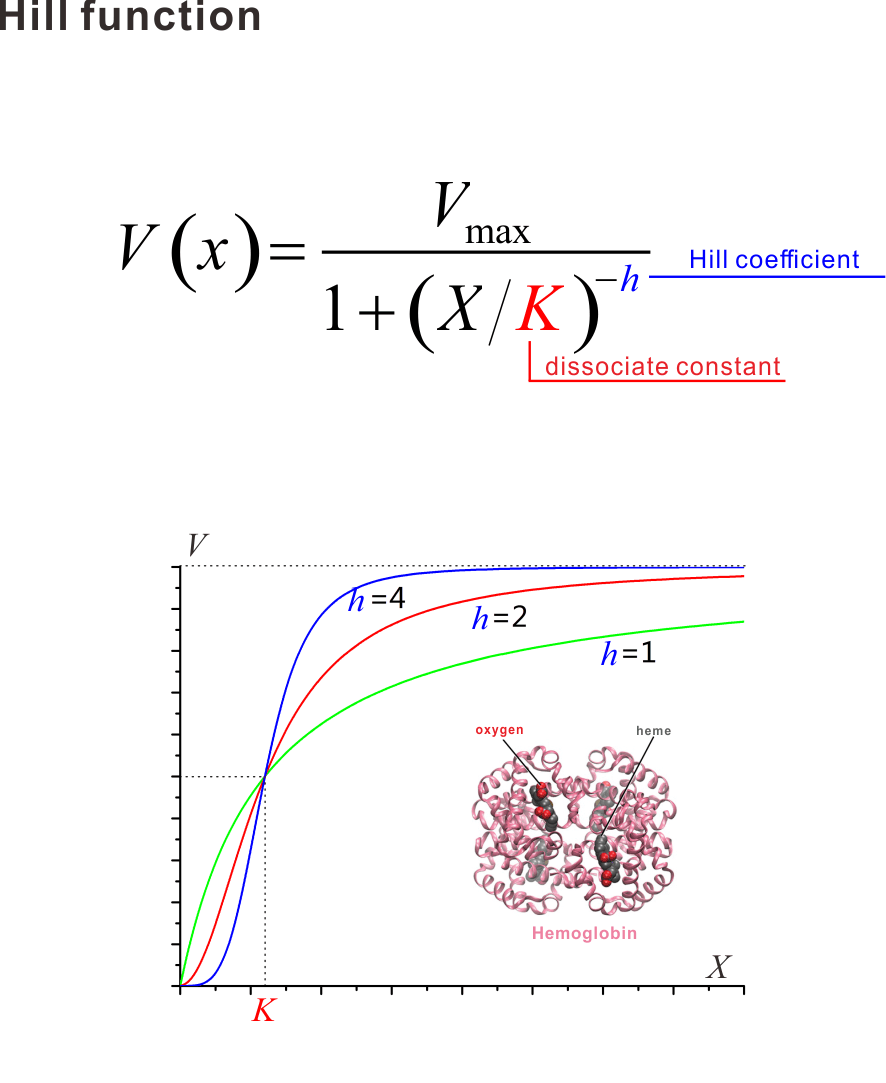


**Figure S****1**. **The Hill function**. The green, red, and blue curves correspond to *h* = 1, 2, and 4, respectively. The inlet shows that a hemoglobin has four binding sites with oxygen; thus, its theoretical Hill coefficient is *h* = 4, corresponding to the blue response curve that is shaper than the green (*h* = 1) and red (*h* = 4) curves.
