# Supplementary material for: A more holistic view of the logarithmic dose–response curve offers greater insights into insulin responses
Source: J Biol Chem. 2024 Nov 29;301(1):108037. doi: 10.1016/j.jbc.2024.108037 (PMC11731574; doi:10.1016/j.jbc.2024.108037)
Supplement: Supplemental Fig. S2 [file mmc2.docx]

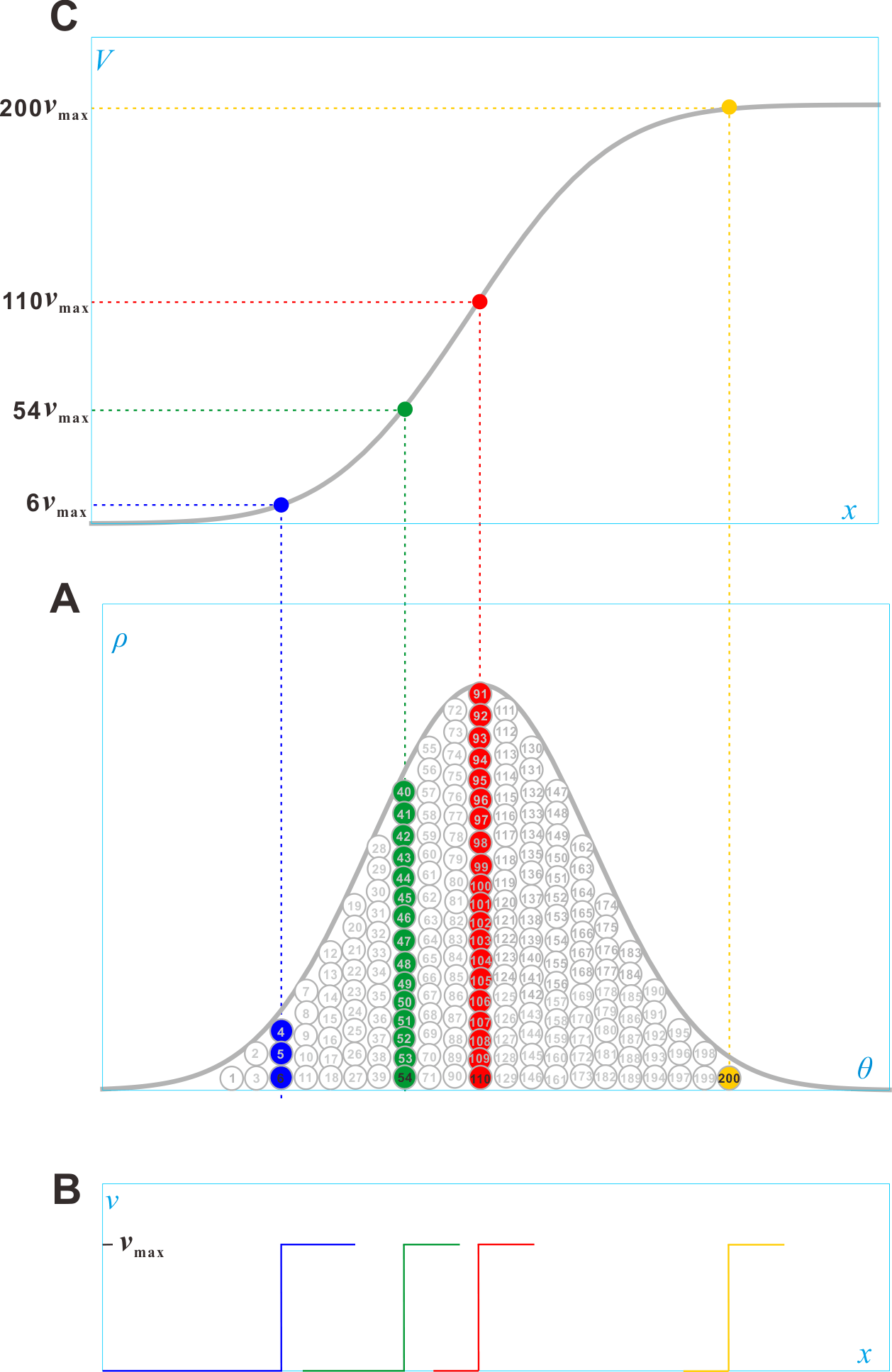


**Figure S****2**. **A more detailed explanation of why the summation of the cells’ all-or-none responses is a graded response at the tissue/organism level**. (**A**) The normal distribution of cells over the log(threshold) values *ρ*(*θ*) (Eq. (7)). The cells are numbered from top to bottom to form a column, and the columns grow from left to right. In this way, the numbering of each bottom cell is precisely equal to the total number of cells to its left or above. Take the vertical green line as an example. Because the numbering of the bottom cell is 54, the total number of cells along or to the left of the vertical green line must be 54. (**B**) The all-or-none response of single cells. The cells have the same *v*_max_ but may have different thresholds; that is, different columns correspond to different *θ* values. (**C**) The log(dose)-response curve *V*(*x*) (Eq. (10)). The value of *V* can be directly read from the cell numbering. Take the green dot as an example: *V* = 54 *v*_max_ because there are 54 cells along or to the left of the vertical green line.
