# Supplementary material for: A more holistic view of the logarithmic dose–response curve offers greater insights into insulin responses
Source: J Biol Chem. 2024 Nov 29;301(1):108037. doi: 10.1016/j.jbc.2024.108037 (PMC11731574; doi:10.1016/j.jbc.2024.108037)
Supplement: Supplemental Fig. S3 [file mmc3.docx]

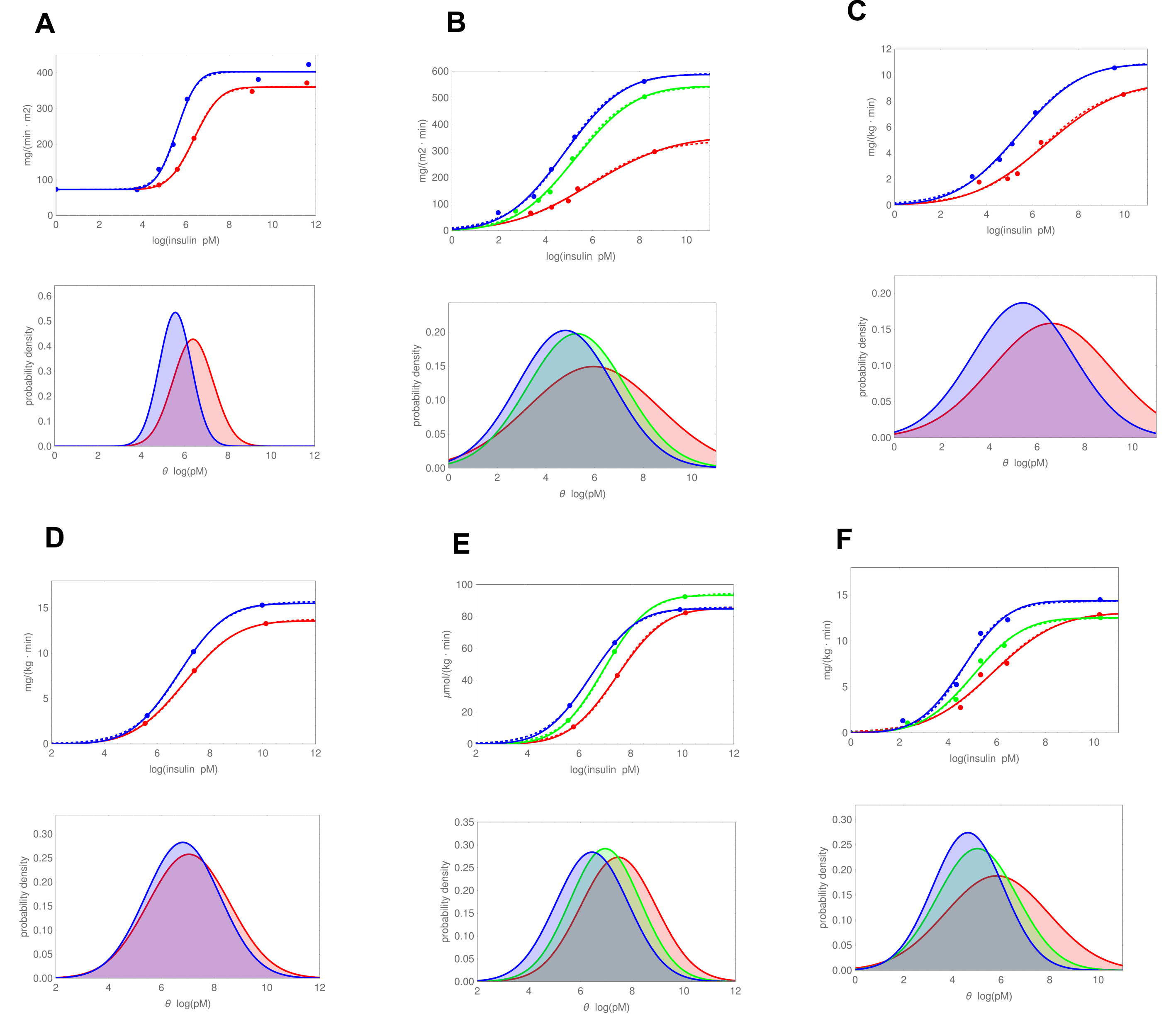


**Figure S****3**. **Insulin-mediated glucose disposal dose responses of human subjects manifesting horizontal shifting (i.e., the adjustability of EC_50_)**. Upper panel: The dots represent data obtained from insulin clamp experiments. The dotted (solid) curves represent the logistic (CND) functions that best fit the data. Lower panel: The threshold distribution *ρ*(*θ*) that underlie the CND functions. (**A**) Research based on the data from [32] of non-obese (blue) and obese (red) subjects. (**B**) Research based on the data from [47] of non-obese (blue), lower-obese (green), and upper-obese (red) subjects. (**C**) Research based on the data from [42] of young (blue) and old (red) subjects. (**D**) Research based on the data from [38] of normotensive (blue) and hypertensive (red) subjects. (**E**) Research based on the data from [39] of lean subjects (blue), hypertensive obese subjects (red), and hypertensive obese subjects after 6 months of aerobic exercises plus weight loss (green). (**F**) Research based on the data from [49] of subjects during rest (red), exercise (blue), and recovery from exercise (green).
