# Supplementary material for: A more holistic view of the logarithmic dose–response curve offers greater insights into insulin responses
Source: J Biol Chem. 2024 Nov 29;301(1):108037. doi: 10.1016/j.jbc.2024.108037 (PMC11731574; doi:10.1016/j.jbc.2024.108037)
Supplement: Supplemental Fig. S4 [file mmc4.docx]

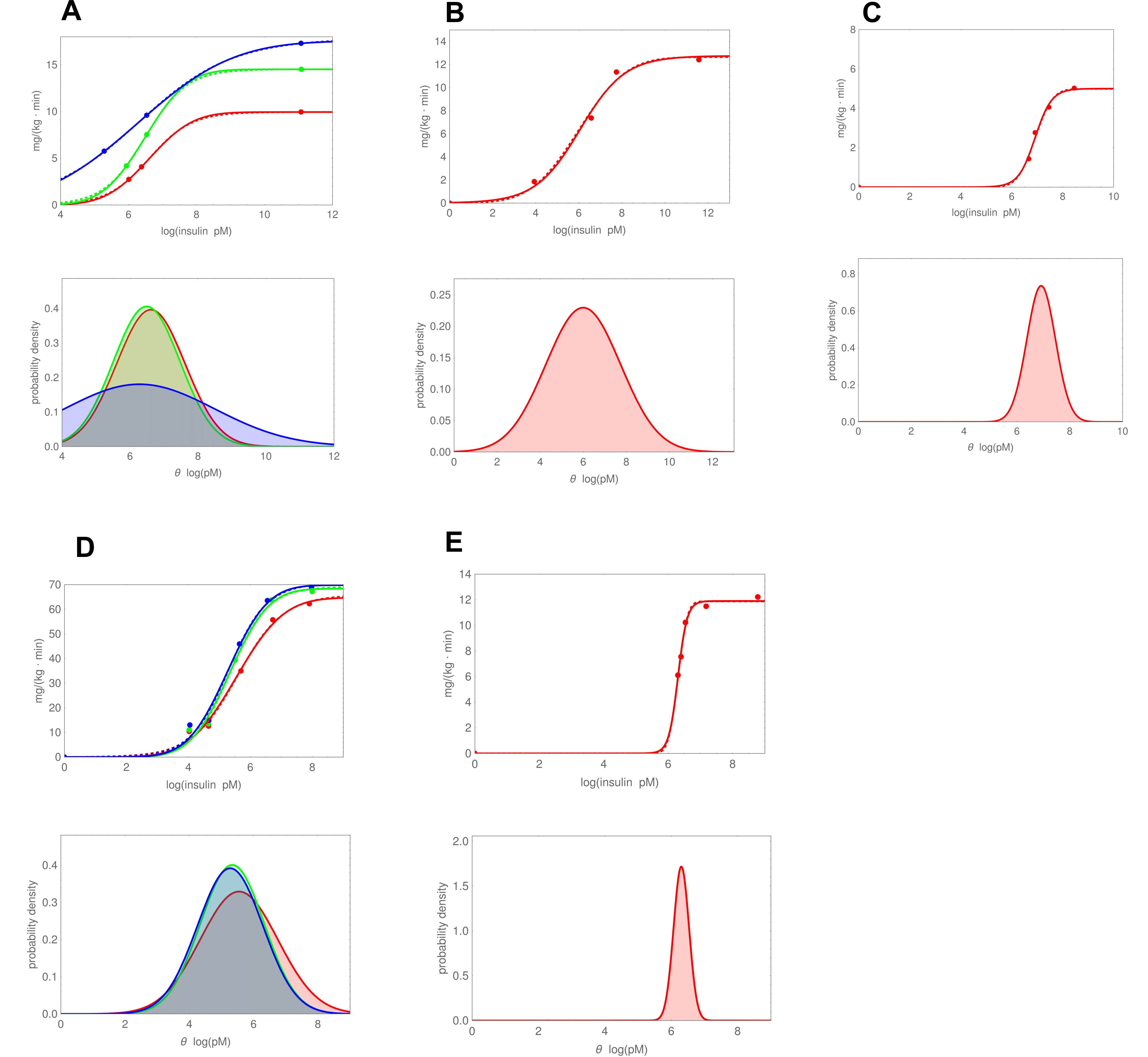


**Figure S****4**. **Insulin-mediated glucose disposal dose responses of human subjects.** (**A, B, C, D, E**) are researches based on the data from [40], [41], [43], [46], [48].
