# Supplementary material for: A more holistic view of the logarithmic dose–response curve offers greater insights into insulin responses
Source: J Biol Chem. 2024 Nov 29;301(1):108037. doi: 10.1016/j.jbc.2024.108037 (PMC11731574; doi:10.1016/j.jbc.2024.108037)
Supplement: Supplemental Fig. S5 [file mmc5.docx]

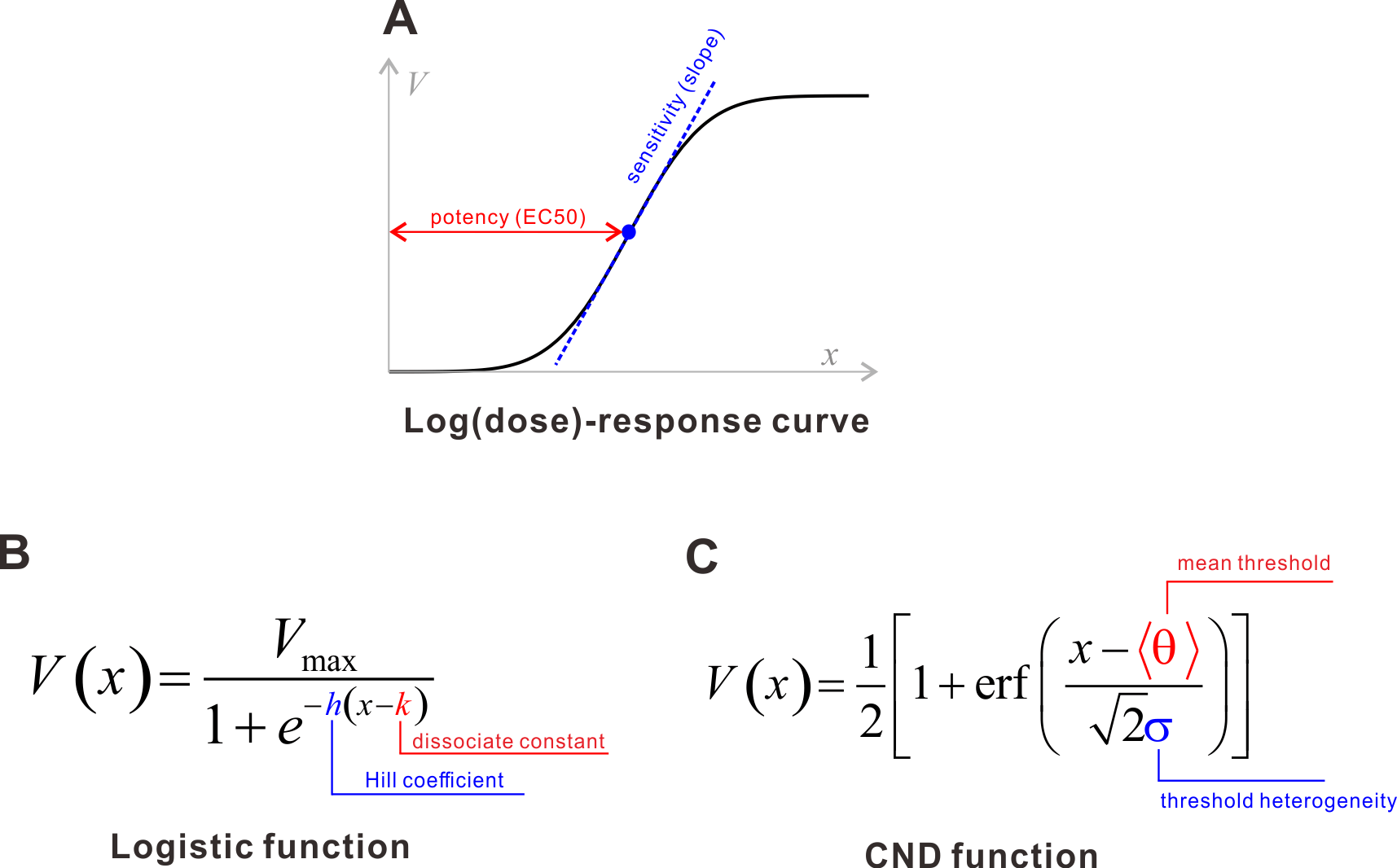


**Figure** **S5. The correspondences between the characteristics of a response curve (A), the parameters of the logistic model (B), and the parameters of the CND model (C)**. The (potency, *k*, <*θ*>) and (sensitivity, *h*, *σ*) correspondence are encoded by red and blue colors, respectively.
